# Impact of 3D technology on perioperative outcomes of articular and periarticular fractures: A systematic review and meta-analysis of RCT studies

**DOI:** 10.1016/j.jor.2026.03.015

**Published:** 2026-03-10

**Authors:** Mattia Alessio Mazzola, Giulia D'Andrea, Niccolo Barducci, Giuseppe Marongiu, Giacomo Placella, Vicenzo Salini

**Affiliations:** a IRCCS Ospedale San Raffaele, Unità Clinica di Ortopedia e Traumatologia, Via Olgettina 60, 20132, Milan, Italy; b Università Vita-Salute San Raffaele, Via Olgettina 58, 20132, Milan, Italy; c Università Degli Studi di Cagliari, Cagliari, Italy

**Keywords:** “3D printing", "Orthopaedic fractures”, “Surgical planning”, “Articular fractures”, “Peri-articular fractures”, “3D modeling”, “Pre-operative planning"

## Abstract

**Background:**

In recent years, three-dimensional (3D) printing and virtual preoperative planning have gained increasing importance in orthopaedic surgery. These innovative technologies for the management of articular and periarticular fractures enable a precise understanding of patient-specific anatomy and accurate preoperative simulations.

**Objectives:**

The objective of this systematic review and meta-analysis is to evaluate and summarize the impact of 3D-assisted techniques on perioperative outcomes in the surgical management of articular and periarticular fractures across different anatomical districts.

**Study design & methods:**

This review and meta-analysis was conducted in accordance with the PRISMA guidelines, and PICOS criteria. Randomized controlled trials (RCTs) investigating the use of 3D technology in the treatment of articular and periarticular fractures were systematically collected and analyzed. Inclusion criteria comprised level I RCTs comparing 3D-assisted surgery (including 3D printing and/or virtual surgical planning) with conventional surgical techniques. Studies were excluded if they were non-randomized or lacked sufficient outcome measures. The primary outcome evaluated was operative time; secondary outcomes included intraoperative blood loss, fluoroscopy duration, and postoperative complication rate.

**Results:**

16 RCTs published between 2015 and 2024 were included, encompassing 881 patients (431 treated with 3D-assisted methods and 450 undergoing conventional surgery). The studies covered several anatomical regions, including tibial plateau, ankle, calcaneus, elbow, and wrist.

Meta-analysis demonstrated that 3D-assisted surgery significantly reduced operative time (tibial plateau: MD = −12.8 min, p < 0.001; ankle: MD = −18.9 min, p < 0.001; elbow: MD = −16.3 min, p < 0.001; wrist: MD = −8.1 min, p < 0.001). Similarly, lower intraoperative blood loss were observed (tibial plateau: MD = −30.4 mL, p < 0.001; ankle: MD = −37.2 mL, p < 0.001; wrist: MD = −15.4 mL, p < 0.001) and fluoroscopy exposure (tibial plateau: MD = −2.1 min, p < 0.001; wrist: MD = −1.1 min, p < 0.001). No statistically significant differences were observed in postoperative complication rates among the analyzed anatomical districts.

**Conclusions:**

The findings of this meta-analysis suggest that the implementation of 3D technology in orthopaedic trauma surgery provides measurable perioperative benefits, including significant reductions in operative time, intraoperative blood loss, and fluoroscopy exposure, without increasing the risk of complications.

## 1 Introduction

Three-dimensional (3D) printing produces tangible objects from digital models through additive manufacturing, standing in contrast to conventional subtractive techniques. This method enables the fabrication of shapes and structures, transforming modern manufacturing.^1^ Although 3D printing offers significant advantages such as high precision and customization, challenges related to cost, production time, and the need for specialized expertise persist ^2^^,^^3^.

In recent years, 3D printing combined with virtual preoperative planning has had a profound impact on orthopaedic surgery, particularly in the management of articular and periarticular fractures.^3^ These technologies facilitate the creation of patient-specific 3D models of fractured bones, allowing surgeons to visualize fracture patterns more accurately, plan surgical strategies, and select appropriate fixation devices 4, 5, 6.

A key benefit is represented by the possibility to design customized implants, including fixation devices for complex fractures, solutions for atypical anatomical regions,^1^^,^^7^ and prosthetic components capable of drug delivery.^8^

Additional applications include “in vitro” surgical simulations on fracture models with customized fixation devices,^9^ patient-specific tools for bone defect reconstruction,^10^ and templates for osteotomies and fixation placement.^11^ Such innovations enhance precision in trauma care and deformity correction. Moreover, 3D printing serves as a valuable communication tool with patients, facilitating understanding of surgical plans, and contributes substantially to residency training through detailed anatomical models.^5^^,^^12^

Nevertheless, despite these technological advances, the true clinical benefits of 3D-assisted surgery remain under discussion. While several studies report gains in surgical efficiency, uncertainties persist regarding long-term functional outcomes, bone healing, and complication rates compared to conventional techniques.

This review aims to present a comprehensive analysis of the current scientific evidence on the application of 3D technology in the surgical treatment of articular and periarticular fractures, highlighting both the advantages and limitations of these emerging innovations.

## 2 Materials and methods

This research was submitted and registered to the international prospective register of systematic reviews, PROSPERO registration number: CRD42024611973.

### 2.1 Criteria for considering studies for this review

This review and meta-analysis exclusively include level I randomized controlled trials (RCTs) comparing 3D-assisted surgery (involving 3D printing and/or 3D virtual planning) with conventional surgical techniques (control group).

Non-randomized trials, cohort and retrospective studies, technical reports, editorial commentaries, non-trauma procedures, as well as ex vivo, biomechanical, preclinical, and clinical studies lacking complete data were excluded.

The evaluated outcomes included operative time (primary outcome), intraoperative blood loss, fluoroscopy time, and complication rates.

### 2.2 Research methods for identification of studies

**Fig. 1:**
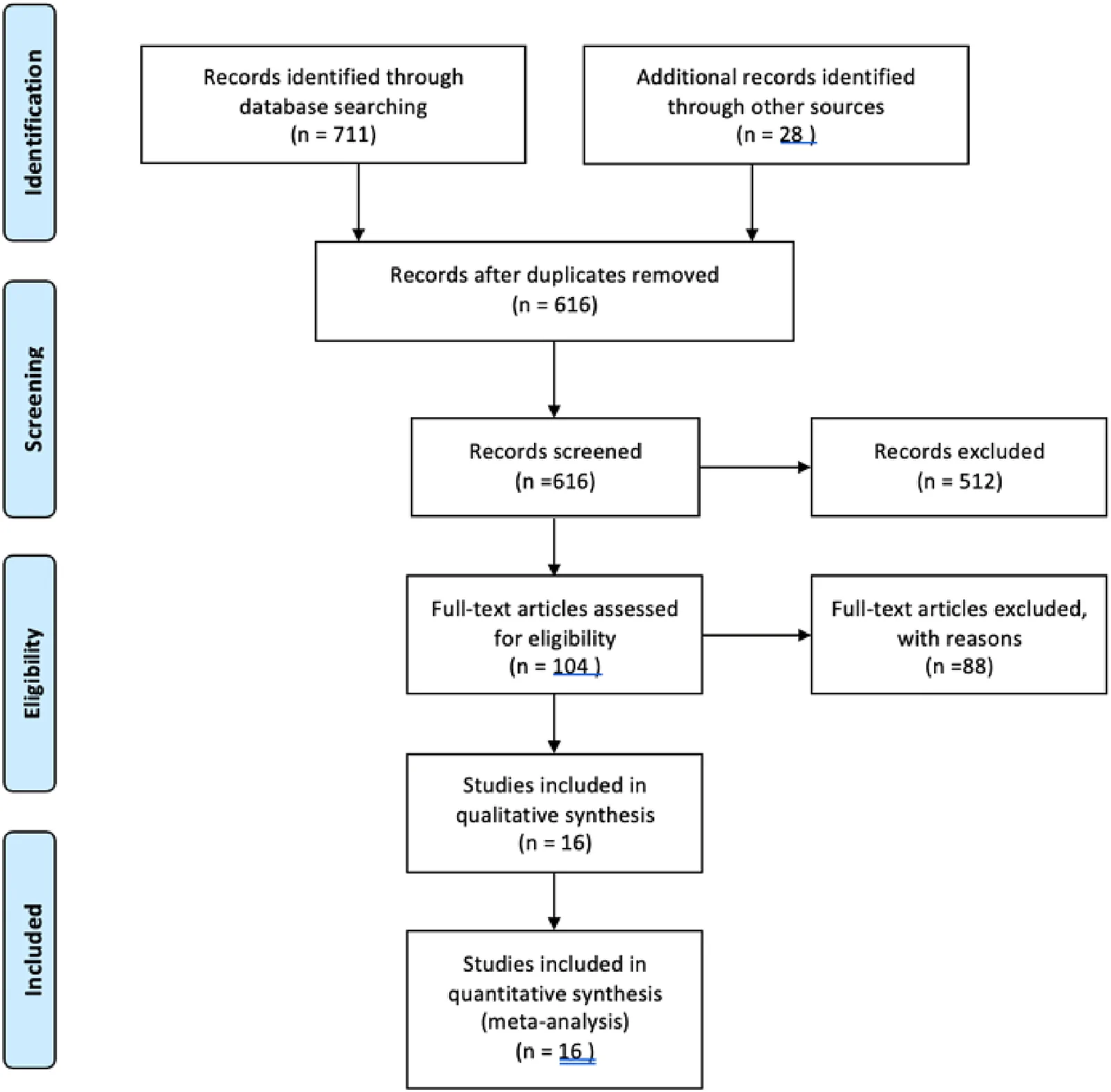
PRISMA representation.

This research was conducted and reported by the methods of the Cochrane Handbook for Systematic Reviews of Interventions and PRISMA guidelines.^13^ A systematic review and meta-analysis of the literature followed the guidelines of the Cochrane Handbook for Systematic Reviews of Interventions and the Preferred Reporting Items for Systematic Reviews and Meta-Analyses (PRISMA) (Fig. 1).

A comprehensive search was performed across multiple databases, including the Cochrane Central Register of Controlled Trials (CENTRAL), MEDLINE/PubMed, Embase, Scopus, Science Citation Index Expanded (Web of Science), ScienceDirect, CINAHL, and LILACS, covering the period from January 1st, 2015, to June 1st, 2024.

The research was conducted using the following keywords alone and in all the various combinations: “3D printing”, “orthopaedic fractures”, “surgical planning”, “articular fractures”, “peri-articular fractures”, “3D modeling”, and “pre-operative planning".

The selection process was based on the participants, intervention, control, outcome, and study design (PICOS).

Two reviewers (BN, GD) independently screened each title and abstract collected from the primary electronic search. In the case of a relevant title and abstract, the full-text version was obtained. All references of each study were accurately screened to search for any additional relevant study potentially missed with the first review process.

The two reviewers independently followed the same checklist to screen all studies and evaluate the eligibility criteria. Disagreements were reviewed by consensus agreement with a third reviewer (MAM).

### 2.3 Data collection and analysis

The level of evidence of included studies was assessed through the adjusted Oxford Centre For Evidence-Based Medicine 2011 Levels of Evidence. The quality of the studies was defined using the Grading of Recommendations Assessment, Development, and Evaluation (GRADE) system,^14^ rating the quality of evidence in systematic reviews (Table 1). After the evidence was collected and summarized, the GRADE system provided explicit criteria for rating the quality of evidence that include study design, risk of bias, imprecision, inconsistency, indirectness, and magnitude of effect.

**Table 1:** GRADE quality evaluation of included studies.

Study	Risk of Bias	Inconsistency	Indirectness	Imprecision	Publication Bias	Overall GRADE
Ozturk et al.	Low	Low	Low	Some Concerns	Low	Moderate
Dai et al.	Low	Low	Low	Low	Low	High
Song et al.	Low	Low	Low	Some Concerns	Low	Moderate
Zhen, Tao et al.	Some Concerns	Low	Low	Low	Some Concerns	Moderate
Yang et al.	Low	Low	Low	Low	Low	High
Zheng, Chen et al.	Some Concerns	Low	Some Concerns	Low	Some Concerns	Moderate
Shen et al.	Low	Low	Low	Low	Low	High
Zhang et al.	Low	Low	Low	Some Concerns	Low	Moderate
Lou et al.	Some Concerns	Low	Low	Low	Some Concerns	Moderate
Zheng, Su et al.	Low	Low	Low	Low	Low	High
Yang et al.	Some Concerns	Low	Low	Low	Some Concerns	Moderate
Shung et al.	Low	Low	Low	Some Concerns	Low	Moderate
Giraldo et al.	Low	Low	Low	Low	Low	High
Kong et al.	Low	Low	Low	Some Concerns	Low	Moderate
Chen et al.	Some Concerns	Low	Low	Low	Some Concerns	Moderate
Chen, Zhou et al.	Low	Low	Low	Low	Low	High

**Fig. 2:**
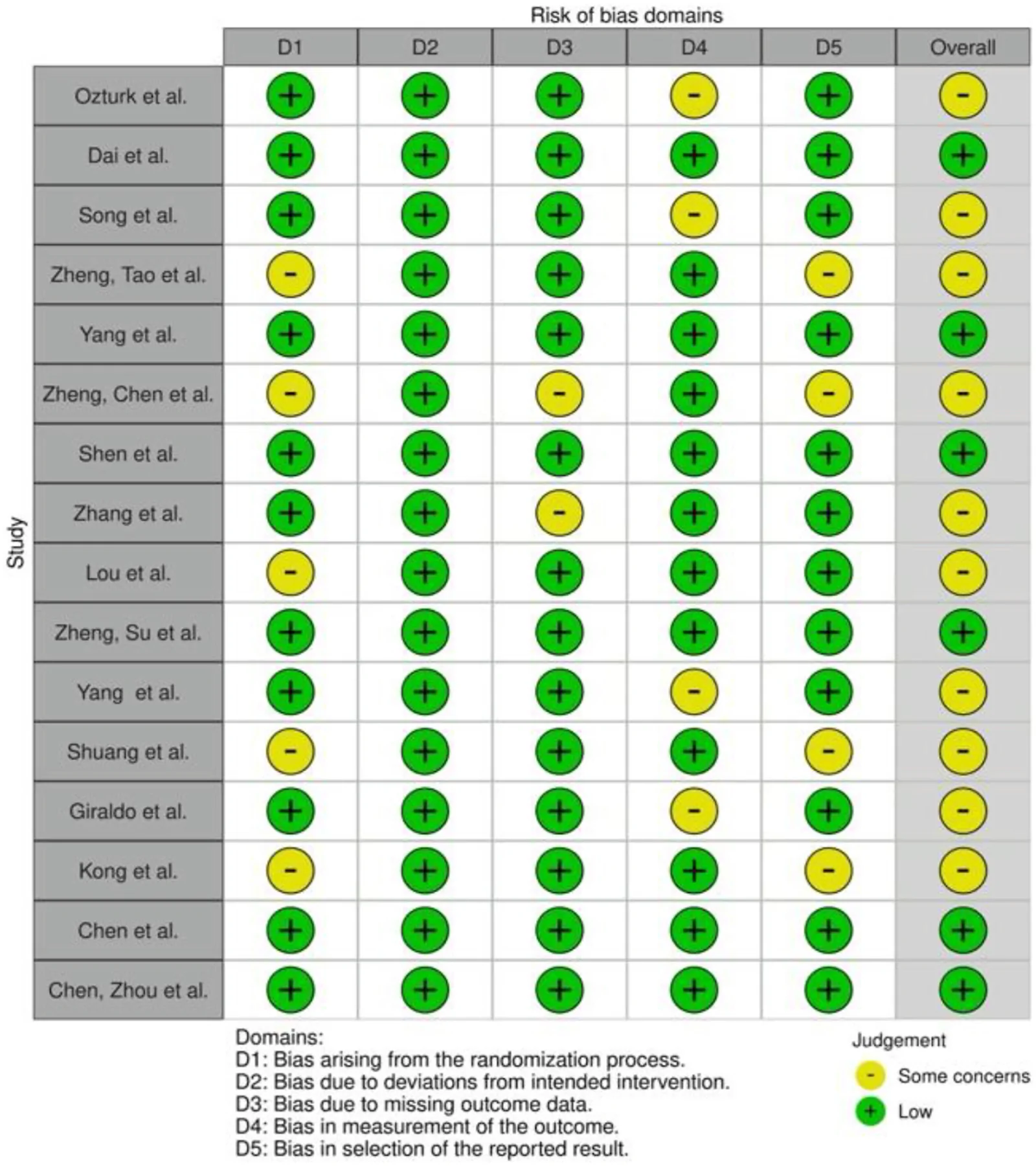
Risk of bias of included studied.

The risk of bias was assessed with the revised tool to assess the risk of bias in randomized trials (RoB 2) and reported in Fig. 2.

RoB 2 is the recommended tool to assess the risk of bias in randomized trials included in Cochrane Reviews. It is structured into a fixed set of domains of bias, focusing on different aspects of trial design, conduct, and reporting. Within each domain, specific questions aim to elicit information about features of the trial that are relevant to risk of bias.

A detailed assessment of study design, level of evidence, year of publication, country, level of evidence, number of participants, study duration, 3D technology used, outcomes and results were independently completed by each reviewer and discrepancies were discussed between the authors.

The analysis was conducted separately for patients who underwent 3D-assisted surgery (study group) and traditional surgery (control group).

Data were extracted and recorded, and general features of each study were summarized in Table 2.

**Table 2:** General features of included studies.

Study	Year	Country	Design	LOE	N	Period	3D Technologies	F-U (M)	Outcome	Result
*Sebastián Giraldo PÁ*	2023	ESP	RCT	II	30 (15 control group – 15 3D group)	2018-2021	3D model printing of fractured wrist; in vitro surgery simulation: fracture and fragment study, reduction and fixation with plate and screws.	6	Surgical time, intra-op scoping time, clinical and radiological results	No significant differences in surgical time, in minute scopes, in the PRWE questionnaire in radiographic parameters with the exception of joint step (P 0.028);
*Kong L.*	2020	CHN	RCT	II	32 (16 control group – 16 3D group)	2017-2018	Surgical simulation on the 3D model using the contralateral wrist as a model: reduction, K-wire placement, volar plate selection and pre-modeling, appropriate screw selection and placement.	6	Surgical time, estimated intra-operative blood loss, number of intra-op scopes; complications; clinical-functional outcomes at 6 months post-op (ROM, VAS, DASH score)	Shorter surgical time, blood loss and intra-op scoping time in the 3D group; no significant differences in post-op complication rates and in clinical-functional parameters
*Chen C*	2019	CHN	RCT	II	48 (25 control group – 23 3D group)	2015	3D virtual image reconstruction; 3D printing of fractured wrist model, fracture study, reduction, K-wire placement, choice of plate and screw type and size.	12	Surgical time, blood loss, intra op scopia time, clinical-functional parameters (modified Gartland-Werley score, ROM), radiological parameters.	In the 3D group less surgical time, intra op scopia time and less blood loss (P < 0.05). No significant differences in clinical-functional and radiological parameters (P > 0.05).
*Chen C.*	2017	CHN	RCT	II	107 (55 control group – 52 3D group)	2013-2015	3D printing of the fracture, choice of plate and screws to be used and in vitro simulation of the operation.	12	Surgical time, blood loss, intra op fluoroscopy time, clinical-functional parameters (Gartland- Werley score, ROM), radiological parameters.	In the 3D group less surgical time, intra op scopia time and less blood loss (P < 0.05). No significant differences in clinical-functional and radiological parameters (P > 0.05).
*Zheng W.*	2018	CHN	RCT	II	91 (48 control group – 43 3D group)	2013-2015	3D model printing used to simulate in vitro surgery: fracture study, planning, reductive manoeuvres, implant choice (plates and screws), plate pre-modeling, screw seat and trajectory; materials then sterilised and implanted to the patient	12	Surgical time, blood loss, intra op scopia time, fracture healing time, clinical-functional parameters (MEPS and DASH score)	Significantly less surgical time, blood loss, number of scopes in the 3D group. No differences in clinical-functional scores or ROM at 1 year between the two groups. No differences in healing time.
*Shuang F.*	2016	CHN	RCT	II	13 (7 control group – 6 3D group)	2014	CT and 3D reconstruction of fractured elbow and healthy elbow; 3D printing of models; in vitro simulation of fracture reduction; 3D printed dedicated plates.	6-13	Surgical time, radiographic bone healing, clinical-functional parameters (ROM, MEPS), complications.	Less surgical time in the 3D group (P < 0.05). No significant differences in clinical-functional parameters between the two groups (P > 0.05). Bone healing in all patients. Complications: 1/7 control group: intraop traction lesion of the ulnar nerve (healed spontaneously at 3 months). No other complications observed.
*Yang L.*	2017	CHN- USA	RCT	II	40 (20 control group – 20 3D group (10 PLA vs. 10 ABS))	2017	CT elbow, 1:1 scale 3D model printing, in vitro simulation of fracture reduction and synthesis Use of two different materials for model printing (PLA vs. ABS).	6-13	Surgical time, blood loss, functional scores (MEPS), anatomical synthesis, differences between the materials used (PLA vs ABS), complications.	3D group vs. control group: shorter surgical time, less blood loss, better functional scores (p < 0.05); no significant differences in the degree of anatomical reduction (p > 0.05). PLA material is more suitable than ABS for 3D model printing.
*Lou Y*	2017	CHN	RCT	ND	72 (38 control group – 34 3D group)	2014-2015	3D virtual model and 3D printed model of tibial plateau fracture; surgical planning: simulation of reduction and stabilization with K wires, choice of fixation devices and modeling of the plates, evaluation of the orientation and location of the screws (fixation device then sterilised and used in surgery)	ND	Surgical time, estimated intra-operative blood loss, number of intra-op scopes; clinical-functional outcomes (HSS score), complications	Shorter surgical time, blood loss and intra-op scoping time in the 3D group; better clinical-funcional score in the 3D group; no significant differences in post-op complication rates
*Zhang H*	2015	CHN	RCT	ND	32 (18 control group – 14 3D group)	2011-2013	3D virtual model of tibial plateau fracture; study of fracture pattern and virtual surgery simulation; implant choise, screw positioning, surgical approach	ND	Surgical time, length of skin incision, estimated blood loss, radiographic parameters	3D group shorter surgical time, less blood loss and shorter skin incision length (P < 0.001) compared to the control group. Post-op radiographic results comparable to pre-op virtual simulation.
*Shen Z*	2023	CHN	RCT	ND	61 (31 control group – 30 3D group)	2015-2021	3D virtual model and 3D printed model of tibial plateau fractures; in vitro surgery simulation, fixation device selection and positioning; subsequently, performed surgery in vivo	18 ± 4	Surgical time, blood loss, number of intra-op fluoroscopy, hospitalization time, bone healing time, radiographic parameters, clinical-functional parameters, complications	3D group shorter surgical time, less blood loss, less number of intraop fluoroscopy compared to the control group (P < 0.05). No significant differences between the two groups regarding hospitalization time, complication rate, clinical-functional results, radiographic parameters (P > 0.05)
*Liang H.*	2023	CHN	Retrospective		48 (24 control group – 24 3D group)	2019-2022	3D fracture model printing, plate selection and printing, screw selection and in-vitro testing	12M	Surgical, fracture reduction and internal fixation time, number of intra-op scopes, total length of surgical incisions, fracture healing time (weeks) fracture reduction degree (Burwell–Charnley criteria), functional score (Mazur score), number of complications.	3D group present less surgical, fracture reduction and internal fixation time, number of intra- op scopes and smaller total length of surgical incisions (p < 0.001). No statistically significant differences between healing time, reduction quality, functional scores and complication rate
*Zheng W.*	2018	CHN	RCT		100 (50 control group – 50 3D group)	2013-2016	Prints of the fracture and contralateral tibia, used for fracture understanding, plate and screw selection and in vitro surgery. The fixation devices and 3D models were sterilised for use during surgery	20M	Surgical time, intra- operative blood loss, scopia time, bone healing time, radiographic parameters (fracture reduction), clinical-functional parameters (ankle ROM, VAS score, AOFAS score)	3D group: significantly less surgical time, less blood loss, less intra-op scopes (P < 0.001); no significant differences in radiographic scores (bone healing time and fracture reduction rate, P > 0.05), in functional scores (plantarflexion and dorsiflexion ROM, VAS score, AOFAS score, P > 0.05) and complications rate.
*Ozturk AM*	2022	TUR	RCT		37 (19 control group – 18 3D group)	2017-2019	virtual and printed 3D model of both calcaneus; pre-operative planning and “in vitro” surgical simulation; plate choice, plate pre-bending, optimal plate position, number, position, orientation and length of screws	15.4	surgical time, blood loss, fluoroscopy number, radiographic parameters, functional parameters (AOFAS), bone healing time	3D group shorter surgical times, lower number of fluoroscopy, lower blood loss compared to the control group (P < 0.0001); functional scores, radiographic parameters and bone healing time no significant differences
*Dai G*	2020	CHN	RCT		81 (41 control group – 40 3D group)	2015-2017	virtual and printed 3D model of calcaneal fracture; in vitro simulation of surgery; determined screw length, trajectory and entry point; quantity and injection site of calcium sulphate cement (CSC)	29.6	Surgical time, blood loss, number of fluoroscopy, radiographic parameters, functional outcomes (AOFAS), complications	Surgical time, blood loss, number of fluoroscopy significantly less in the 3D group. Time from injury to surgery longer in the 3D group. 3D group significantly better X-ray results compared to the control group except for the heel width. AOFAS score 3D group significantly higher.
*Song Q*	2023	CHN	RCT		32 (13 control group – 19 3D group)	2020-2022	3D virtual model of calcaneus fracture; design and 3D printing of customized cast- mask with guides for K-wire insertion (as a guide for cannulated screws); sterilization of the printed model and use in surgery	≥6	Surgical time, number of fluoroscopy, hospitalization time, radiographic and clinical- functional parameters (AOFAS), complications	3D group significantly shorter surgical time, number of fluoroscopy and hospitalization time, better AOFAS score, better some radiographic parameters (heel width and height, Bohler angle; no significant differences in heel length and Gissane angle
*Zheng W*	2017	CHN	RCT		75 (40 control group – 35 3D group)	2014-2016	3D model printing of fractured calcaneus; in vitro simulation of surgery; pre-bending of plates and choice of screws	12	Surgical time, blood loss, number of fluoroscopy, fracture consolidation time, Bohler angle, Gissane angle, heel height and width	3D group significant shorter surgery time, less blood loss, less number of fluoroscopy; no significant differences in fracture healing time, clinical-functional and radiographic parameters

### 2.4 Statistical analysis

The effect of 3D-assisted surgery in periarticular and articular fractures was tested using the inverse variance method and the data were expressed as the mean differences (MD) and 95% CI for continuous measures. For dichotomous variables, the Mantel–Haenszel test was used, and the data were expressed as risk ratios (RR) and 95% CI.

Statistical heterogeneity among the studies was assessed using the *χ*2 test and I2, using I2 > 40% as the cut-off value to indicate a significant heterogeneity. A fixed-effects model was adopted when I2 < 40%, otherwise, a random-effects model was used. When standard deviations were not available from the full-text articles, they were calculated using the method described in the Cochrane Handbook for Systematic Reviews of Interventions.^15^

Quantitative synthesis was performed for all the outcomes that were reported in at least three studies.

## 3 Results

### 3.1 Elbow fixation

#### 3.1.1 Operative time

**Fig. 3:**
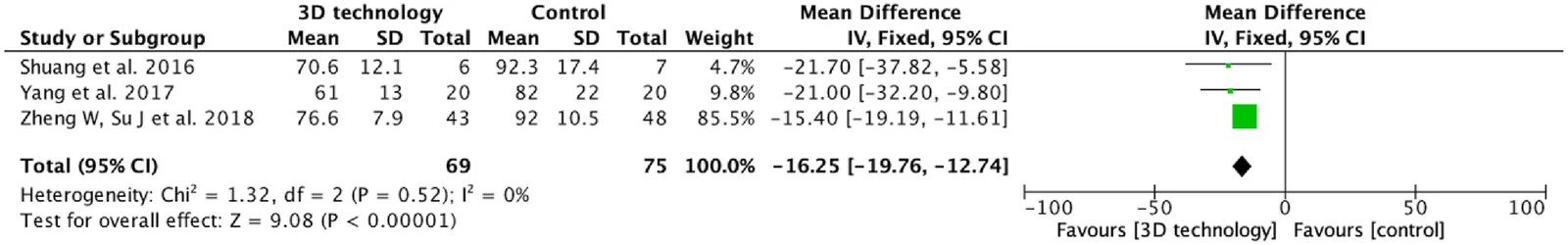
Forest plot of the operative time in elbow fixation.

Operative time in elbow fixation was measured in 3 RCTs 16, 17, 18 involving 144 patients (69 in the 3D group and 75 in the control group). All studies showed a statistically significant improved operative time for the 3D group. Meta-analysis demonstrated that 3D technology significantly improved operative time with a mean difference of −16.3 (95%CI: 19.8 to −12.7) minutes (I^2^ = 0%, p < 0.001). Fig. 3 shows the forest plot for the operative time in elbow fixation.

#### 3.1.2 Blood loss

Blood loss was measured only in 2 RCT studies ^16^^,^^17^ involving 131 patients (63 in the 3D group and 68 in the control group). All these studies showed a statistically significant decrease in blood loss for the 3D group. Meta-analysis was not performed for the paucity of studies.

#### 3.1.3 Complications

**Fig. 4:**
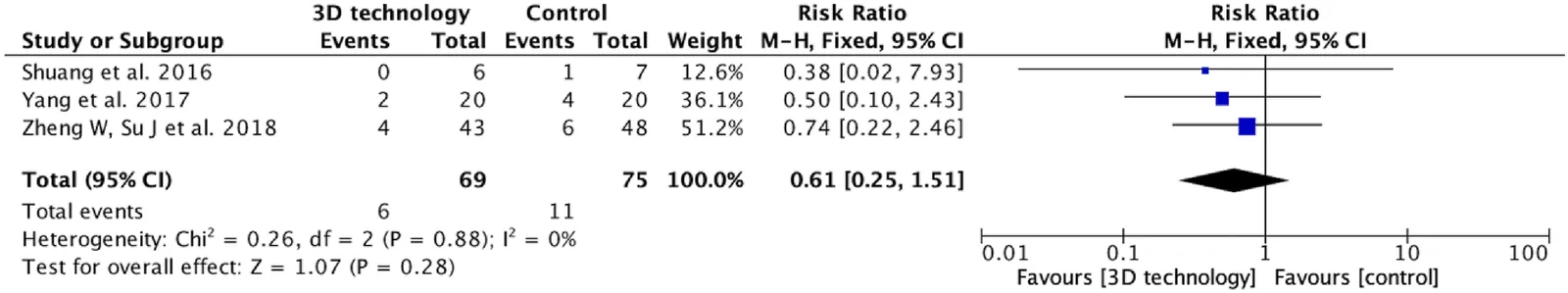
Forest plot of complication rate in elbow fixation.

Complications were measured in 3 RCTs 15, 16, 17 involving 144 patients (69 in the 3D group and 75 in the control group). All studies showed no difference in complication rate among groups and meta-analysis confirmed no significant differences in complication rate, although patients in 3D group had lower complication rate (8.7% for the 3D group and 14.7% in control group) (I^2^ = 0%, p = 0.28). Fig. 4 shows the forest plot for the complication rate in elbow fixation.

### 3.2 Wrist fixation

#### 3.2.1 Operative time

**Fig. 5:**
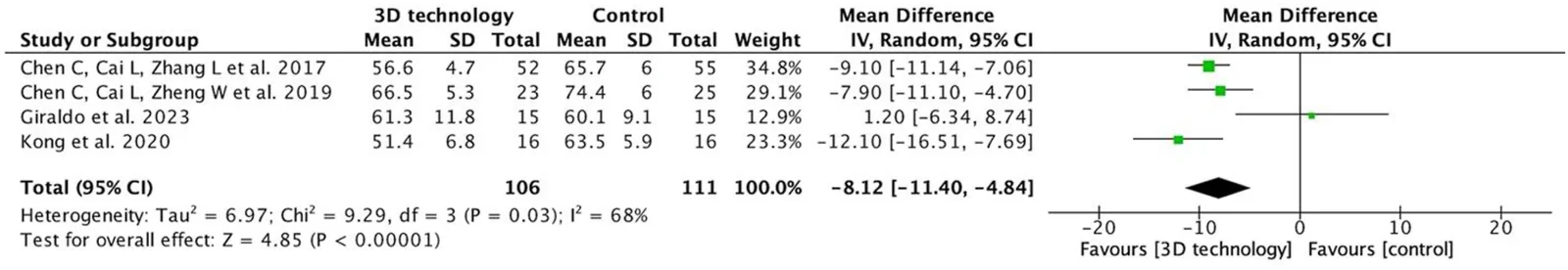
Forest plot of operative time in wrist fixation.

Operative time in wrist fixation was measured in 4 RCTs ^11^^,^^18^, ^19^, ^20^, involving 217 patients (106 in the 3D group and 111 in the control group). All studies, except Giraldo et al.,^19^ showed a statistically significant improved operative time for the 3D group. Meta-analysis demonstrated that 3D technology significantly improved operative time with a mean difference of −8.1 (95%CI: 11.4 to −4.8) minutes (I^2^ = 68%, p < 0.001). Fig. 5 shows the forest plot for the operative time in wrist fixation.

#### 3.2.2 Fluoroscopy time

**Fig. 6:**
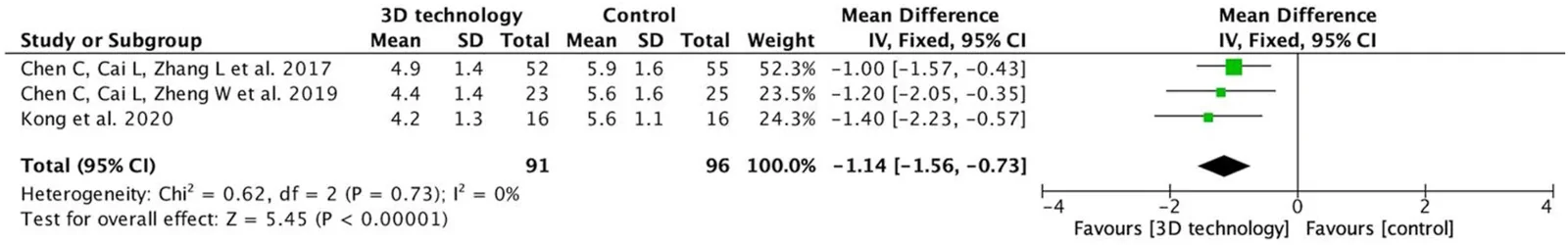
Forest plot of fluoroscopy time in wrist fixation.

Fluoroscopy time in wrist fixation was measured in 3 RCTs ^11^^,^^18^^,^^20^ involving 187 patients (91 in the 3D group and 96 in the control group). All studies showed a statistically significant decrease in exposure to fluoroscopy in the 3D group. Meta-analysis demonstrated that 3D technology significantly decreased exposure to fluoroscopy with a mean difference of −1.1 (95%CI: 1.6 to −0.7) minutes (I^2^ = 0%, p < 0.001). Fig. 6 shows the forest plot for the fluoroscopy time in wrist fixation.

#### 3.2.3 Blood loss

Blood loss in wrist fixation was measured in 3 RCTs ^11^^,^^18^ involving 187 patients (91 in the 3D group and 96 in the control group). All studies showed a statistically significant improvement in blood loss for the 3D group. Meta-analysis demonstrated that 3D technology significantly decreased blood loss with a mean difference of −15.4 (95%CI: 19.7 to −11.1) mL (I^2^ = 59%, p < 0.001).

### 3.3 Tibial plateau

#### 3.3.1 Operative time

**Fig. 7:**
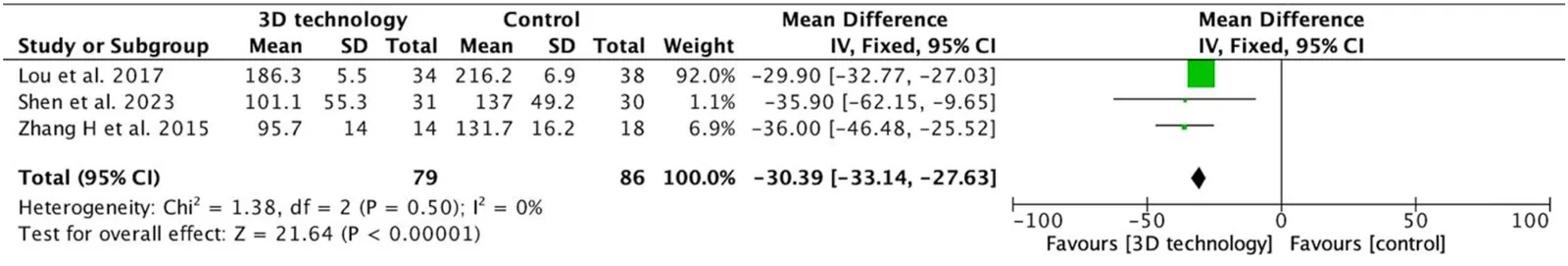
Forest plot of operative time in tibial plateaus fixation.

Operative time in tibial plateau fixation was measured in 3 RCTs 21, 22, 23 involving 165 patients (79 in the 3D group and 86 in the control group). All included studies demonstrated decreased operative time in the 3D group. Meta-analysis showed that 3D technology significantly reduced operative time with a mean difference of −30.4 (95%CI: 33.1 to −27.6) min (I^2^ = 0%, p < 0.001). Fig. 7 shows the forest plot for the operative time in tibial plateau fixation.

#### 3.3.2 Fluoroscopy time

Fluoroscopy exposure was measured only in 2 RCT studies ^22^^,^^23^ involving 133 patients (65 in the 3D group and 68 in the control group). All these studies showed a decrease in fluoroscopy exposure for the 3D group. Meta-analysis was not performed for the paucity of studies.

### 3.4 Complications

Complications were reported in only in 2 RCT studies ^22^^,^^23^ involving 133 patients (65 in the 3D group and 68 in the control group). The complication rate was comparable between groups with a 7.7% complication rate in the 3D group and 13.2% in the control group. Meta-analysis was not performed for the paucity of studies.

### 3.5 Ankle

#### 3.5.1 Operative time

Operative time in ankle fixation was measured in only 2 RCTs ^24^^,^^25^ involving 130 patients (65 in the 3D group and 65 in control group). The lack of studies precluded a meta-analysis. All studies reported a statistically significant reduction in operative time for the 3D group.

#### 3.5.2 Blood loss

Blood loss in ankle fixation was measured in only 2 RCTs ^24^^,^^25^ involving 130 patients (65 in the 3D group and 65 in control group). The lack of studies precluded a meta-analysis. All studies reported a statistically significant reduction in blood loss for the 3D group.

### 3.6 Calcaneus

#### 3.6.1 Operative time

**Fig. 8:**
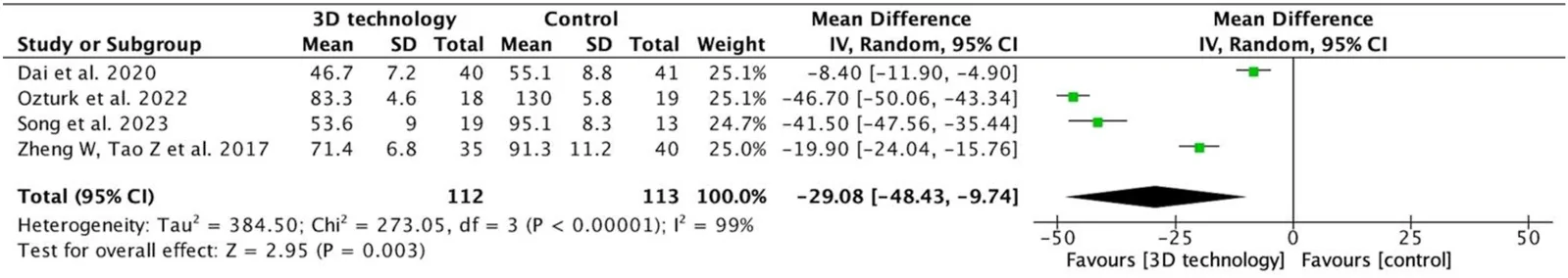
Forest plot of operative time in calcaneus fixation.

Operative time in calcaneus fixation was measured in 4 RCTs 26, 27, 28, 29 involving 225 patients (112 in the 3D group and 113 in the control group). All included studies demonstrated decreased operative time in the 3D group. Meta-analysis showed that 3D technology significantly reduced operative time with a mean difference of −29.1 (95%CI: 48.5 to −9.7) min (I^2^ = 99%, p = 0.003). Fig. 8 shows the forest plot for the operative time in calcaneus fixation.

#### 3.6.2 Fluoroscopy time

**Fig. 9:**
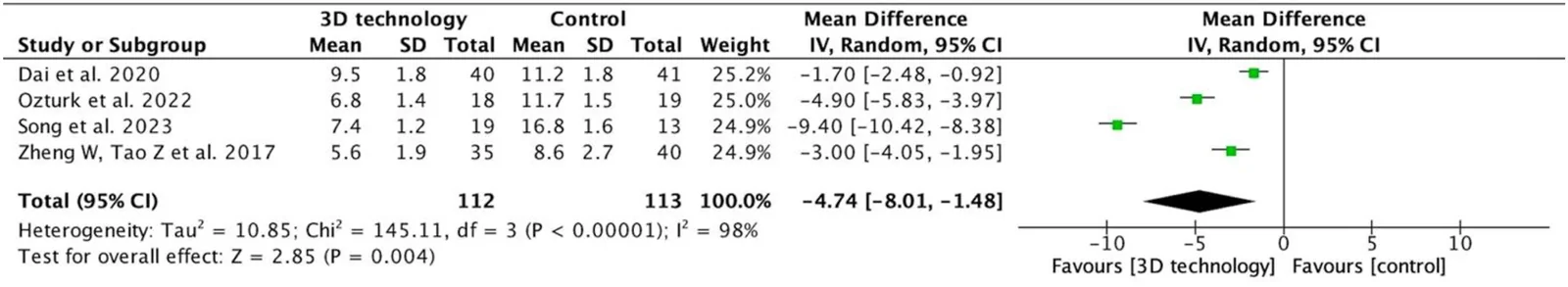
Forest plot of fluoroscopy time in calcaneus fixation.

Fluoroscopy time in calcaneus fixation was measured in 4 RCTs 26, 27, 28, 29 involving 225 patients (112 in the 3D group and 113 in the control group). All studies showed a statistically significant decrease in exposure to fluoroscopy in the 3D group. Meta-analysis demonstrated that 3D technology significantly decreased exposure to fluoroscopy with a mean difference of −4.74 (95%CI: 8.0 to −1.5) minutes (I^2^ = 98%, p = 0.004). Fig. 9 shows the forest plot for the fluoroscopy time in calcaneus fixation.

#### 3.6.3 Blood loss

**Fig. 10:**
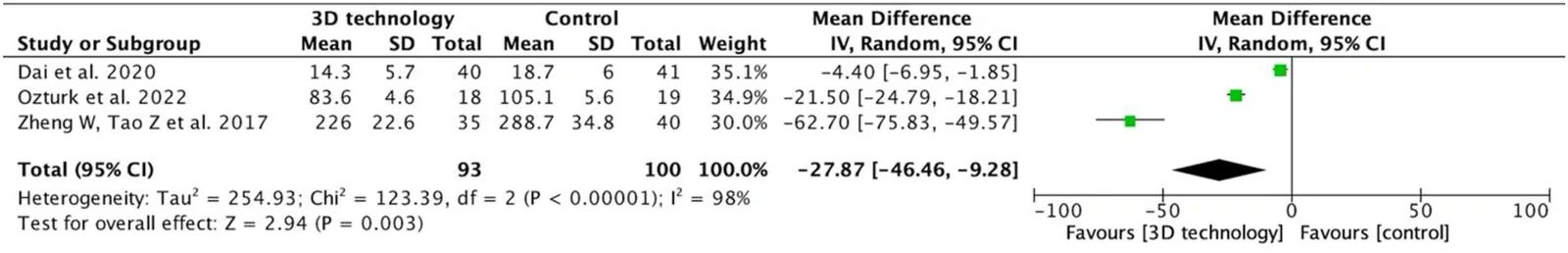
Forest plot of blood loss in calcaneus fixation.

Blood loss in calcaneus fixation was measured in 3 RCTs ^26^^,^^27^^,^^29^ involving 193 patients (93 in the 3D group and 100 in the control group). All studies showed a statistically significant improvement in blood loss for the 3D group. Meta-analysis demonstrated that 3D technology significantly decreased blood loss with a mean difference of −27.9 (95%CI: 46.5 to −9.3) mL (I^2^ = 98%, p = 0.003). Fig. 10 shows the forest plot for the blood loss in calcaneus fixation.

### 3.7 Complications

**Fig. 11:**
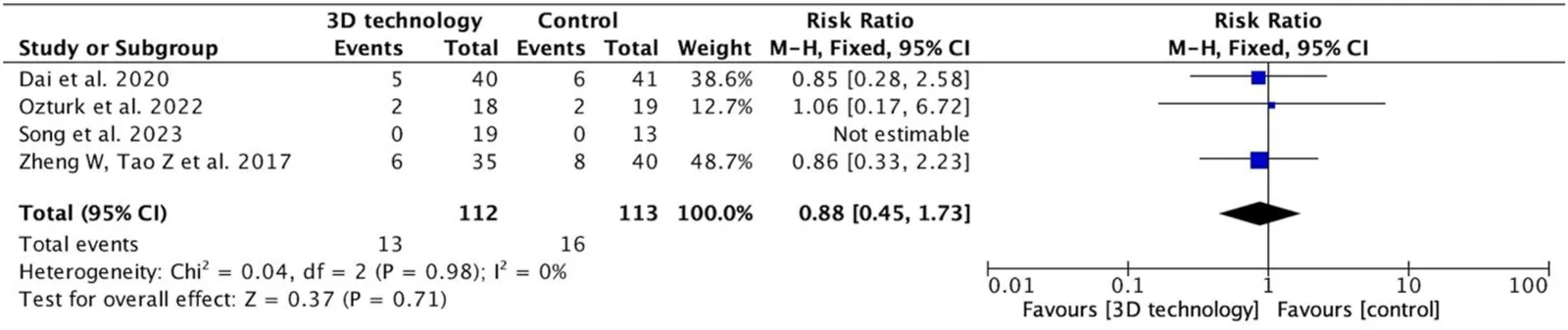
Forest plot complication rate in calcaneus fixation.

Complications were measured in 4 RCTs 26, 27, 28, 29 involving 225 patients (112 in the 3D group and 113 in the control group). All studies showed no difference in complication rate among groups and meta-analysis confirmed no significant differences in complication rate among groups (11.6% for the 3D group and 14.2% in control group) (I^2^ = 0%, p = 0.71). Fig. 11 shows the forest plot for the complication rate in calcaneus fixation.

## 4 Discussion

In recent years, orthopaedic surgery has undergone significant technological advancement, among which the introduction of three-dimensional (3D) printing represents one of the most promising innovations, particularly in the management of articular and periarticular fractures. The main goal of fracture surgery is to restore limb function, relieve pain, and achieve stable fixation and anatomical correction of deformities. The use of 3D printing technology facilitates preoperative study of the fracture, allowing surgeons to better understand the fracture pattern before surgery. This is especially beneficial in complex fractures, where 3D visualization of specific anatomical areas helps the surgeon plan the reduction more accurately, leading to improved intraoperative precision and better anatomical reconstruction.

Moreover, this technology enables the design of patient-specific fixation devices that conform to the individual's anatomy, improving implant fit and stability. It also assists in determining screw length, incision location, and orientation, thereby contributing to a safer and more controlled surgical procedure.^30^

A multicentric study conducted by Bagaria and Chaudhary^7^ demonstrated the crucial role of 3D-printed models in complex orthopaedic surgeries, particularly for articular and periarticular fractures. The authors showed that these models enhance preoperative planning and procedural simulation, offering a more comprehensive understanding of fracture morphology compared to conventional imaging. Consequently, 3D-assisted approaches improve reduction accuracy, shorten surgical time, and enhance operative precision.

Similarly, Upex et al.^31^ supported the use of 3D printing for preoperative planning, reporting a significant improvement in the quality of fracture reduction and a reduction in operative duration.

Across the randomized controlled trials included in this review, 3D-assisted surgery consistently shortened operative time compared with traditional techniques, with reductions ranging from approximately 8 min in wrist fixation ^11^^,^^18^, ^19^, ^20^ to nearly 30 min in tibial plateau and calcaneal fractures 21, 22, 23^,^^26^, ^27^, ^28^, ^29^. These results confirm that 3D technology enhances surgical precision through patient-specific anatomical visualization and preoperative simulation, allowing surgeons to analyze fracture morphology and optimize fixation strategies with greater accuracy.

Another important finding of this meta-analysis concerns intraoperative blood loss, which was consistently reduced in 3D-assisted groups, as reported by Yang et al.,^17^ Chen et al.,^11^ and Dai et al..^26^ This reduction is attributable to the improved understanding of fracture anatomy and the precise positioning of implants, which minimize unnecessary dissection and manipulation.

Similarly, fluoroscopy exposure was significantly decreased in wrist ^11^^,^^18^^,^^20^, calcaneal 26, 27, 28, 29, and tibial plateau fractures ^22^^,^^23^, supporting that 3D-assisted approaches improve spatial orientation and reduce the need for repeated intraoperative imaging.

Importantly, no significant differences in postoperative complication rates were observed among the included studies ^11^^,^^16^, ^17^, ^18^, ^19^, ^20^, ^21^, ^22^, ^23^, ^24^, ^25^, ^26^, ^27^, ^28^, ^29^. Comparable complication incidences (7–14%) between 3D-assisted and conventional groups indicate that the use of 3D printing and virtual planning does not compromise surgical safety.

Giannopoulos et al.^32^ also demonstrated that 3D printing models enhance diagnostic accuracy, facilitate preoperative planning, and help surgeons assimilate complex anatomical information more rapidly, confirming their potential as valuable tools in surgical education and future clinical practice.

The application of 3D printing continues to expand, and in the near future it is expected to play an even greater role in the production of customized implants and prosthetic materials.^33^ Currently, its uses extend from the creation of physical bone models and patient-specific tools to surgical guide development, assisting orthopaedic surgeons in dealing with complex cases with greater precision and confidence.

The findings of this meta-analysis further strengthen previous evidence supporting the perioperative advantages of 3D-assisted techniques in orthopaedic trauma.

Earlier studies by Bizzotto et al.^6^^,^^8^ and Bagaria et al.^7^ highlighted the benefits of 3D printing for preoperative planning and surgical training, though quantitative data were limited.

By focusing exclusively on level I randomized trials, the present analysis provides stronger evidence that 3D-assisted planning translates into measurable improvements in surgical efficiency. The most significant benefits were observed in anatomically complex regions such as the tibial plateau and calcaneus: Lou et al.^21^ and Shen et al.^22^ demonstrated that 3D-guided tibial plateau fixation significantly reduced operative time and improved reduction accuracy, while Ozturk et al.^27^ and Dai et al.^26^ showed that 3D-printed templates improve screw trajectory planning and reduce intraoperative corrections in calcaneal fractures.

In upper limb surgery, Yang et al.^17^ and Zheng et al.^18^ reported similar perioperative advantages in intercondylar humeral fractures, with shorter operative times and reduced blood loss compared to standard procedures.

Collectively, these studies support the hypothesis that 3D technology is particularly valuable in anatomically demanding regions where fracture morphology is difficult to assess with conventional imaging alone.

From a clinical perspective, 3D printing and virtual preoperative planning serve as valuable adjuncts to traditional orthopaedic trauma surgery. They allow surgeons to anticipate intraoperative challenges, improve fixation accuracy, and optimize implant selection, while also enhancing patient communication and resident education.^5^^,^^11^^,^^12^

However, certain limitations continue to restrict widespread clinical adoption, such as the cost and time required for model fabrication and the need for technical expertise. These challenges are expected to diminish as faster, more affordable printing technologies and automated software become widely available, making 3D-assisted planning more feasible in routine practice.

Despite the inclusion of high-level RCTs, this review is subject to limitations, including heterogeneity in fracture types, surgical techniques, and 3D printing modalities, as well as small sample sizes in some subgroups such as ankle ^24^^,^^25^ and tibial plateau 21, 22, 23 fractures.

Future research should focus on long-term outcomes, including bone healing, functional recovery, and cost-effectiveness, while developing standardized 3D workflow reporting and learning curve assessment.

## 5 Conclusion

In conclusion, despite the limitations of this study, which include the analysis of different anatomical districts and the use of various 3D modeling techniques, the inclusion of high-level randomized controlled trials provides robust and reliable evidence. The results demonstrate that the use of 3D printing technology in the surgical treatment of articular and periarticular fractures is associated with a significant reduction in operative time and intraoperative blood loss, without an increase in postoperative complications. These advantages can be attributed to the use of 3D technology to study fracture morphology preoperatively, thereby improving intraoperative accuracy and surgical planning. Furthermore, the creation of patient specific 3D models enables more precise and stable fracture reduction. Future studies should focus on long-term functional and radiological outcomes to better evaluate the lasting clinical benefits of 3D assisted surgical techniques.

## Guardian/patient's consent

None Needed.

## Ethical approval

This study is a systematic review of existing published data and does not involve human participants or identifiable personal data. Therefore, ethical approval was not required in accordance with institutional guidelines.

## Credit author statements

Mattia Alessio Mazzola: Conceptualization; Methodology; Formal analysis; Writing – Original Draft; Writing – Review & Editing.

Giulia D'Andrea: Investigation; Data curation; Writing – Original Draft; Writing – Review & Editing.

Niccolo’ Barducci: Investigation; Data curation; Writing – Original Draft; Writing – Review & Editing.

Giuseppe Marongiu: Investigation; Data curation; Writing – Original Draft; Writing – Review & Editing.

Giacomo Placella: Investigation; Methodology; Supervision; Writing – Review & Editing.

ViNcenzo Salini: Investigation; Supervision; Writing – Review & Editing.

## Funding statement

This research did not receive any specific grant from funding agencies in the public, commercial, or not-for-profit sectors.

## Declaration of competing interest

None.
